# Species and Strain Differences in the Susceptibility of the Cervico-Vaginal Squamous Epithelium to 3,4-Benzo(a)pyrene

**DOI:** 10.1038/bjc.1965.50

**Published:** 1965-06

**Authors:** M. Thiery, M. van Gijsegem


### SPECIES AND STRAIN DIFFERENCES IN THE SUSCEPTIBILITY OF THE CERVICO-VAGINAL SQUAMOUS EPITHELIUM TO 3,4-BENZO(A)PYRENE

M. THIERY AND M. VAN GIJSEGEM

*From the Department of Obstetrics and Gynaecology of the University of Ghent, Belgium*

Received for publication December 7, 1964

ON purely speculative grounds it would seem that a treatment as aggressive as painting of the mouse portio with a concentrated suspension of a rapidly effective (von Euler and Skarzynski, 1942) and potent (Iball, 1939) carcinogenic compound like 3,4-benzo(a)pyrene would very soon induce carcinoma in all the experimental animals, but experimental investigation has shown that this is not the case. Indeed, the results of a pilot study indicate that, in the inbred strains of mice used, there are marked differences in the neoplastic response of this epithelium. This observation applies to an even greater degree to other rodent species (Thiery, 1963*a*).

The literature fails to supply the data required for comparison of the degree of susceptibility of the cervico-vaginal epithelium of various rodent species to benzo(a)pyrene. The authors who report having induced carcinoma of the uterine cervix in the mouse by chemical means, used widely differing carcinogenic substances and applied them by even more dissimilar techniques. Mutual comparison of their results therefore is futile. Authors who report on painting experiments of the mouse cervix with benzo(a)pyrene used almost exclusively C3H-mice (von Haam and Scarpelli, 1955 ; Koprowska *et al.*, 1958). Only von Haam and Alberty (1960) have compared several mouse strains in this respect, but to the best of our knowledge their study has not been published. Using endocervical insertion of methylcholanthrene-impregnated threads, they found differences in susceptibility of the cervico-vaginal epithelium in C3H, BDF-1, Swiss, DBA, and A mice. Working independently of these authors and using a different technique, we arrived at analogous conclusions (Thiery, 1963*a*).

Fusco (1932) induced a single squamous cell carcinoma by painting the vaginal walls of rats with coal tar. Only two groups of authors have used pure carcinogens in experiments with this species. They used a thread technique (Vellios and Griffin, 1957) or painted the portio (Glucksmann, 1956 ; Glucksmann and Cherry, 1958 ; Cherry and Glucksmann, 1960) with dimethylbenzanthracene (DMBA). The first group succeeded in inducing squamous cell carcinoma of the portio, vagina, and vulva.

Wachtel (1956) attempted unsuccessfully to provoke carcinoma in the hamster by painting the uterine cervix with methylcholanthrene. Using benzo(a)pyrene, we were able to provoke squamous cell carcinoma of the vagina and portio in this species (Thiery, 1963*a*, *b*).

We have been unable to find any data in the literature concerning the chemical inducement of cervico-vaginal tumours in the guinea-pig or in *Mastomys natalensis.*

The need for comparable data has led us to make further investigations into the difference in neoplastic response of the cervico-vaginal squamous epithelium to benzo(a)pyrene. To enable semi-quantitative analysis of our results, the methods used were standardized as strictly as possible.

### MATERIALS AND METHODS

Twelve different strains of inbred mice‡ were studied. All the animals were 2- to 2½-month-old virgins. The rats, guinea-pigs, Syrian hamsters, and *Mastomys natalensis* were of course hybrids. Numbers of animals treated, strains, and substrains are listed in Table I. Housed under standard conditions, the animals were fed a commercial pelleted diet with water *ad libitum*. Twice weekly the portio of

TABLE I.—*Species and Strains Used for the Induction of Cervico-vaginal Carcinoma*

| Species | Strain | Substrain | Number | Origin |
|---|---|---|---|---|
| Mouse (*Mus musculus*) | C3H | N | 451 | Nat. Inst. of Health, Bethesda, Md., USA |
| | A | Jax | 9 | Jackson Memorial Laboratory, Bar Harbor, Maine, USA |
| | 0·20 | A | 12 | Antoni Van Leewenhoekhuis, Amsterdam, The Netherlands |
| | N | TNO | 25 | Centraal Proefdierenbedrijf, T.N.O., Austerlitz, The Netherlands |
| | R | TNO | 25 | ,, |
| | K | TNO | 23 | ,, |
| | Q | TNO | 26 | ,, |
| | Mo | TNO | 25 | ,, |
| | S | A | 38 | Antoni Van Leeuwenhoekhuis, Amsterdam, The Netherlands |
| | AKR | A* | 12 | ,, |
| | H | TNO | 25 | Centraal Proefdierenbedrijf, T.N.O., Austerlitz, The Netherlands |
| | WLL | A† | 12 | Antoni Van Leeuwenhoekhuis, Amsterdam, The Netherlands |
| Rat (*Rattus norwegicus*, var. alba) | — | — | 11 | Centraal Proefdierenbedrijf, T.N.O., Austerlitz, The Netherlands |
| Syrian Hamster (*Mesocricetus auratus*) | — | — | 12 | ,, |
| Guinea-pig (*Cavia cobaya*) | — | — | 6 | ,, |
| *Mastomys natalensis* | — | — | 15 | Station d'Acclimatation et d'Elevage, Bouillé-Saint Paul, France |

* This substrain no longer shows leukaemia in the 20th inbred generation.
† The milk factor is absent in this substrain ; in contrast to the strain with the milk factor, the incidence of mammary carcinoma is very low here.

‡ Abbreviations used for inbred strains of mice and their substrains are as recommended by the Commission on Inbred Strain Nomenclature (Carter *et al.*, 1952). Because the Centraal Proefdierenbedrijf (T.N.O., Austerlitz, the Netherlands) is not included in the list, we have used the symbol TNO for animals obtained from this Institute.

the animals was examined through an otoscope with a built-in light source (National Electric Instrument Co., Ltd.) and painted with a 1% suspension of 3,4-benzo(a)pyrene in acetone. As soon as a tumour could be detected by the eye or a mass palpated through the abdominal wall, the animal was killed and a complete post-mortem examination performed. The genital tracts as well as any organ showing gross pathology were removed for study. Tissues were fixed in formaldehyde and processed in the usual manner. All tissues were stained with haematoxylin and eosin.

### RESULTS

Multiple epithelial lesions were found in close proximity to each other in the genital tracts of the treated animals. We suppose that the chemical canceration of the squamous epithelium covering the vaginal walls, the portio, and the cervical canal of the mouse may be viewed as a chain reaction (Thiery, 1963*a*). The earliest epithelial lesion takes the form of hyperkeratosis, and may depend upon the so-called oestro-mimetic action of 3,4-benzo(a)pyrene (Thiery, 1963*a*). This type of epithelial pathology is followed successively by dysplasias of increasing intensity, *in situ* carcinoma, incipient carcinoma characterized by early stromal invasion and, finally, squamous cell carcinoma. For each experimental animal only the most advanced epithelial lesion is recorded here. The incidence of cervico-vaginal squamous cell carcinoma induced in the mouse is given in Table II. The overall incidence of cervico-vaginal tumours induced in the mouse as well as in the other rodents is listed in Table III.

TABLE II.—*Incidence of Cervico-vaginal Squamous Cell Carcinoma Induced in Mice by Painting the Cervix with Benzo(a)pyrene*

| Strain | Number of animals* A | Number of animals* B | Number of cervico-vaginal tumours† | Number of paintings |
|---|---|---|---|---|
| C3H | 451 | 379 | 376 | 40 |
| A | 9 | 7 | 7 | 23–40 |
| 0·20 | 12 | 10 | 10 | 38–77 |
| N | 25 | 23 | 23 | 31–100 |
| R | 25 | 25 | 25 | 39–91 |
| K | 23 | 23 | 21 | 35–43 |
| Q | 26 | 24 | 20 | 37–85 |
| Mo | 25 | 19 | 14 | 36–46 |
| S | 38 | 31 | 18 | 55–77 |
| AKR | 12 | 10 | 5 | 67–78 |
| H | 25 | 19 | 3 | 64–92 |
| WLL | 12 | 10 | 1 | 63 |

* A : painted ; B: used for the histo-anatomical study.
† Including incipient squamous cell carcinoma.

### *The Mouse*

*C3H-strain.*—Of 451 painted C3H mice, 379 were suitable for histoanatomical study.

The histogram (Fig. 1) shows the correlation between the most advanced epithelial lesion and the time factor (number of paintings, henceforth abbreviated as p). The time is given in intervals of 5 p or 2·5 weeks.

Hyperkeratosis, as the most advanced epithelial change in the genital tract under study, is found in the interval of 1–10 p (Fig. 1). Dysplasia makes its

TABLE III.—*Overall Incidence of Cervico-vaginal Tumours Induced in Laboratory Rodents by Painting the Cervix with Benzo(a)pyrene*

| Species | Number of animals necropsied | Number of cervico-vaginal squamous cell carcinomas* | Number of cervico-vaginal sarcomas |
|---|---|---|---|
| Mouse | 580 | 523 | 3 |
| Rat | 10 | 0 | 2 |
| Hamster | 10 | 9 | 0 |
| Guinea-pig | 4 | 0 | 0 |
| *Mastomys* | 10 | 5 | 2 |

* Including incipient squamous cell carcinoma.

appearance equally soon after the initiation of treatment (Fig. 1), but in contrast to hyperkeratosis remains present over a long period. The incidence of this epithelial lesion increases with the number of paintings, reaches a maximum, and decreases rapidly thereafter. This can be clearly seen from the histogram (Fig. 2)

FIG. 1.—Histogram showing the incidence of the various lesions induced in the cervico-vaginal squamous epithelium of the C3H mouse. Time in number of paintings.

N—Normal

H—Hyperkeratosis

D—Dysplasia

ISC—*In situ* carcinoma

IC—Invasive carcinoma

FIG. 2.—Histogram showing the relative incidence of dysplasia induced in the cervico-vaginal epithelium of the C3H mouse. Time in number of paintings.

showing the relative incidence of dysplastic epithelium for the various time intervals : the incidence of dysplasia increases following the first treatment, amounts to almost 100 % after 10–15 p, drops rapidly between 16 and 25 p, and is quite rare after 26 p. Intra-epithelial carcinoma was seen very early (5–10 p) several times. The incidence of *in situ* carcinoma as the most highly developed epithelial lesion was about 25 % between 15 and 30 p (Fig. 3). The earliest squamous cell carcinoma was found in C3H mice after 15–20 p (Fig. 4). The incidence of

FIG. 3.—Histogram showing the relative incidence of *in situ* carcinoma induced in the cervico-vagina epithelium of the C3H mouse. Time in number of paintings.

FIG. 4.—Histogram showing the relative incidence of cervico-vaginal squamous cell carcinoma induced in the C3H mouse. Time in number of paintings.

such tumours increases rapidly up to 35–40 p, where the relative frequency amounts to practically 100 %. All the chemically-provoked carcinomas were of the squamous cell type, mostly highly differentiated ; 18 % were mucoepidermoid carcinomas. In 6 % of the mice cervico-vaginal carcinoma showed metastasis, either in the lungs (11 times) or in the ovary (once). The regional lymph nodes of all tumour-bearing mice were examined in serial sections. Metastasis was found in 18 %, with the following localizations : Lnn. lumbales (14 times), Lnn. lumbales and sacrales (7 times), Lnn. lumbales and inguinalis superficialis (once), Ln. inguinalis superficialis (once), and Ln. sacralis (once).

The following coincidental lesions were found : cystoma ovarii simplex (once) ; serous cyst of the tube (once) ; teratoma ovarii solidum (twice) ; mucometra (2 %) ; pyometra (6 %) ; pyosalpinx (1 %) ; abscesses in the primary tumour (15 %) ; purulent peritonitis (2 %) ; adhesions between internal genitalia and abdominal walls (9 %) ; liver abscesses (2 %) ; and lung abscesses (7 %). The incidence of mammary adenocarcinoma is 3 %. Splenomegaly is very frequent in tumour-bearing animals.

*A-strain.*—Nine animals were painted, of which 7 were suitable for histo-anatomical investigation.

All the animals (7/7) developed squamous cell carcinoma following 23–40 p.

Coincidental pathology : mammary adenocarcinoma (once).

0·20-*strain.*—Twelve animals were included in the study and 10 were autopsied and histologically studied.

All (10/10) showed squamous cell carcinoma after 38–77 p.

Coincidental lesions : adenocarcinoma mammae (twice).

*N-strain.*—Twenty-five animals were painted, 23 were suitable.

Epithelial lesions : squamous cell carcinoma (22 times after 31–91 p) ; carcinoma incipiens (once after 100 p). Four of the 22 tumours were mucoepidermoid carcinomas.

Coincidental lesions : pulmonary adenoma (3 times after 50–58 p) ; mucometra (once after 58 p) in a mouse with a large cervico-vaginal carcinoma ; cystoma ovarii simplex (once).

*R-strain.*—Twenty-five animals were painted ; all were suitable for histo-anatomical study.

Squamous cell carcinoma was induced (39–91 p) in all the mice ; one of these (1/25) was a mucoepidermoid carcinoma.

Coincidental lesions : mucometra (twice after 75–76 p in mice with large tumours) ; pyometra (twice after 72 and 80 p) ; bilateral hydronephrosis (twice after 41 and 75 p) ; ovarian abscess (once after 91 p).

*K-strain.*—Twenty-three animals were painted ; all were suitable for study.

Epithelial lesions : squamous cell carcinoma (20 times after 35–43 p), of which 5 (5/20) were mucoepidermoid carcinomas ; carcinoma incipiens (once after 40 p), and dysplasia (twice after 43 p). In one mouse with mucoepidermoid carcinoma, metastatic deposits were found in both lungs, but no mucus cells could be identified.

*Q-strain.*—Twenty-six animals were included in the experiment, 24 of which were suitable for study.

Epithelial lesions : squamous cell carcinoma (18 times after 37–85 p) ; carcinoma incipiens (twice after 54 p) ; *in situ* carcinoma (once after 100 p) ; and dysplasia (3 times after 42–54 p).

Coincidental lesions : adenocarcinoma mammae (5 times after 42–91 p) ; bilateral hydronephrosis (once) in an animal with an enormous cervical tumour and parametrial invasion extending to the pelvic walls ; multiple liver abscesses (once) ; pulmonary adenoma (once after 54 p) ; and hydrosalpinx (once).

*Mo-strain.*—Twenty-five animals were painted ; 19 were suitable. The surviving animals had to be killed after 46 p because of an infection in our colony.

Epithelial lesions : squamous cell carcinoma (12 times after 36–46 p), 2 of which were mucoepidermoid carcinomas ; carcinoma incipiens (twice after 40 p) ; *in situ* carcinoma (3 times after 43–46 p) ; and dysplasia (twice after 11 and 44 p). One squamous cell carcinoma gave rise to metastasis in the lumbar lymph nodes.

Coincident pathology : multiple abscesses of the wall of the small intestine (twice after 46 p).

*S-strain.*—Of 38 treated animals, 31 were suitable for study.

Epithelial lesions : squamous cell carcinoma (16 times after 55–68 p), of which 5 were mucoepidermoid carcinomas ; carcinoma incipiens (twice after 68 and 77 p) ; *in situ* carcinoma (once after 77 p) ; and dysplasia (8 times after 12–77 p). In 3 animals the epithelium was normal after 6–47 p ; 1 showed necrosis after 7 p. In 2 mice, in addition to a squamous cell carcinoma, a sarcoma of the vaginal wall and portio (2/16) was found.

*AKR-strain.*—Twelve animals were painted and 10 used in the investigation.

Epithelial lesions : squamous cell carcinoma (3 times after 67–78 p) ; carcinoma incipiens (twice after 78 p) ; dysplasia (5 times after 78 p).

Coincidental lesions : adenoma of the lung (4 times after 67–78 p).

*H-strain.*—Twenty-five animals were painted ; 19 of them were suitable for the investigation. All surviving mice were killed after 94 p.

Epithelial lesions : squamous cell carcinoma (3 times after 64–92 p) ; dysplasia (3 times after 85–94 p) ; hyperkeratosis (11 times after 85–94 p) ; atrophic (once after 44 p) and necrotic (once after 94 p) epithelium ; sarcoma of the vaginal wall (once after 74 p).

Coincidental lesions : mucometra (once after 94 p) and pyometra (8 times after 94 p). One mouse with hyperkeratotic cervico-vaginal epithelium (85 p) showed multiple adenomas of the lung. In another animal with the same type of epithelium (94 p) we found adenocarcinoma in addition to multiple adenomatous foci. The liver showed multiple areas of adenocarcinoma, probably metastases of a primary lung tumour.

*WLL-strain.*—Of the 12 animals included in the study, 10 were suitable.

Epithelial lesions : mucoepidermoid carcinoma (once after 63 p) ; dysplasia (7 times after 6–42 p) ; hyperkeratosis (twice after 6 and 9 p).

Coincidental lesions : cystoma ovarii simplex (once after 6 p).

*The rat*

Eleven 3–4-month-old virgins were painted, of which 10 were suitable for study. The surviving animals were killed after 173 p.

No cervico-vaginal squamous cell carcinoma was observed. After 105–173 p, the genital squamous epithelium was usually atrophic (7/10) ; in 3 animals it showed a normal appearance after 173 p. Two rats (125 and 144 p) showed a sarcoma of the vaginal wall. These tumours, which measured 28 × 15 × 13 mm. and 15 × 15 × 10 mm. respectively, had a necrotic surface partially covered with atrophic squamous epithelium. The histological picture was of small-cell, poorly-differentiated sarcomas whose histogenesis could not be further clarified.

Coincidental lesions : lung abscess (7 times after 105–173 p) and broncho-pneumonia (once after 125 p).

*The hamster*

Of 12 3–4-month-old virgin hamsters, 10 were suitable for the investigation.

Epithelial lesions : squamous cell carcinoma (8 times after 46–52 p) 3 of which were mucoepidermoid carcinomas ; incipient squamous cell carcinoma (once after 51 p) ; and *in situ* carcinoma (once after 52 p).

Coincidental lesions : cystoma ovarii simplex (once) and peritonitis (twice).

*The cavia*

Of 6 3-month-old guinea pigs, 2 died after 6 p ; the remaining 4 were killed after 63 p. The cervico-vaginal epithelium of these 4 animals showed only discrete dysplasia.

Coincidental lesion : pyometra (once).

*Mastomys natalensis*

Fifteen animals were painted and 10 used in the study.

Epithelial lesions : squamous cell carcinoma of the portio and vagina (5 times after 66–96 p), 2 of which were mucoepidermoid carcinomas, dysplasia (4 times after 49–96 p) and normal epithelium in prooestrus (once after 96 p). Two animals developed sarcoma of the vaginal wall ; on one of them the cervico-vaginal epithelium was dysplastic (81 p), the other also showed squamous cell carcinoma of the portio (66 p).

Coincidental lesion : adenoma of the lung (once after 60 p).

### DISCUSSION

A standardized method proved successful in provoking squamous cell carcinoma of the portio and/or vagina in almost all C3H mice in less than 20 weeks. The results make it clear, however, that not all mouse strains are equally susceptible to such carcinogenic influence. If the effect of carcinogenesis is evaluated by its two classic parameters (tumour yield and length of induction period), the cervico-vaginal epithelium of 8 strains (C3H, A, 0·20, N, R, K, Q, and Mo) is found to be highly sensitive to benzo(a)pyrene, that of 2 strains (S and AKR) to be moderately sensitive, and that of 2 other strains (H and WLL) to show little sensitivity (Table II). No conclusions could be drawn concerning WLL mice, however, because they had to be killed within too short a period.

The mouse thus shows distinct variations according to strain in the sensitivity of the cervico-vaginal squamous epithelium to locally administered benzo(a)-pyrene. We were able to demonstrate analogous differences in other rodents with respect to the neoplastic response of this epithelium (Table III). In this sense, with the method applied in this investigation, the genital squamous epithelium of rats and guinea-pigs was found to be completely resistant and the hamsters to react very rapidly. The latter finding is in agreement with the results obtained by Chu, Herrold and Wood (1962). The contradictory results of others (Wachtel 1956) are probably to be ascribed to genetic differences among the hamsters used. *Mastomys natalensis*, a rodent which occupies, phylogenetically, an intermediate place between the mouse and the rat, reacts to benzo(a)pyrene partly as a mouse (squamous cell carcinoma) and partly as a rat (sarcoma). Caviae show no sensitivity to painting with benzo(a)pyrene. It is indeed known that the epidermis of this species fails to react to locally-applied carcinogenic substances (benzo(a)-pyrene : Oberling *et al.*, 1936 ; methylcholanthrene : Silberstein and Silberstein,

1947; DMBA: Berenblum, 1949) and that spontaneous cervico-vaginal carcinoma is unknown in it (Tamaschke, 1955).

All the cervico-vaginal epithelial tumours provoked by us in the mouse with benzo(a)pyrene have been squamous cell carcinomas. A histological variant, mucoepidermoid carcinoma, comprised 18% of the total number of tumours provoked in C3H mice. Mucoepidermoid carcinoma was also observed in 6 other mouse strains (N, R, K, Mo, S, and WLL). The cumulative incidence (18/96) agrees with that of the C3H mice. Although the histogenesis of this type of tumour remains obscure, there are indications that sex steroids may play a role (Glucksmann and Cherry, 1962). Rodents whose cervico-vaginal epithelium reacts to benzo(a) pyrene (hamster and *Mastomys*) develop, like the mouse, mucoepidermoid carcinomas. The series studied in this investigation are too small, however, to permit the drawing of conclusions concerning the relative frequency of this special type of tumour.

Adenocarcinoma was not observed in the genital tracts of treated mice. The obvious resistance of genital glandular epithelium to benzo(a)pyrene came somewhat as a surprise to us because we definitely know that benzo(a)pyrene is resorbed from the cervix and acts as a weak oestrogenic compound on the cervico-vaginal as well as on the uterine glandular epithelium.* It is suggested, therefore, that the lack of susceptibility of glandular epithelium is a species characteristic in the mouse because it has been described in extragenital sites also (endometrium: Korteweg and Wijsenbeek, 1933; colon: Thomas, 1935, 1936).

Pan and Gardner (1948), who induced tumours of the mouse portio by inclusion of a small crystal of methylcholanthrene in transplanted cervices, observed adenocarcinoma and sarcoma in addition to squamous cell carcinoma. This result may perhaps bear some relation to the technique employed.

Connective tissue tumours are only exceptionally induced in the mouse by benzo(a)pyrene: the histo-anatomical study of 580 animals showed only 3 cases of sarcoma. Furthermore, these tumours were limited to strains in which the cervico-vaginal epithelium produced only a slight reaction to benzo(a)pyrene (S and H). The localization of the sarcomas was cervico-vaginal in 2 cases and vaginal in 1 case. Two animals also developed squamous cell carcinoma of the cervix. Our results are in agreement with the general impression obtained from the literature that chemically-induced sarcoma of the vagina and/or cervix is relatively rare in the mouse (Glucksmann and Cherry, 1962: 1/98; Murphy, 1961: 22/479; Wachtel, 1961: 2/17) or even absent in series of appreciable size (Scarpelli and von Haam, 1957; von Haam and Scarpelli, 1955; Koprowska *et al.*, 1958; Bogacz and Koprowska, 1961; Reagan, Wentz and Machicao, 1955). In the rat, a species which in our study was found to be resistant to benzo(a)-pyrene, vaginal sarcomas were not rare (2/10). The yield, although relatively small, is nonetheless highly significant because spontaneous sarcoma of the female genital tract is extremely rare in the rat (Tamaschke, 1955). The resistance of the cervico-vaginal epithelium of the rat to benzo(a)pyrene is the more remarkable because spontaneous squamous cell carcinoma with this localization has been reported to be less infrequent than in the mouse (Bullock and Curtis, 1930).

* Four days after a single visual treatment of the portio of ovariectomized C3H mice with 1% benzo(a)pyrene in acetone, the structure of the epithelium of the vagina and endometrium does not differ from that found during spontaneous oestrus (Thiery, 1963*a*). This offers sufficient proof of the fact that the oestromimetic action of benzo(a)pyrene is not limited to the squamous genital epithelium.

Indeed, this lack of neoplastic response of the cervico-vaginal epithelium to benzo(a)pyrene corresponds to that of the epidermis (Glucksmann, 1945), although it would not be correct to state that the epidermis of the rat is completely resistant to local treatment with benzo(a)pyrene (Oberling and Guerin, 1947). The rat epidermis is much more sensitive to DMBA (Berenblum, 1949), which may explain why Glucksmann (1956) was able to induce carcinoma of the vulva with this carcinogen. According to some authors (Vellios and Griffin, 1957), the vaginal epithelium is sensitive to DMBA ; according to others (Cherry and Glucksmann, 1960), only slightly so. It is interesting, however, that the sensitivity to DMBA of both the vaginal epithelium and the vaginal connective tissue can be modified by oophorectomy or the administration of sex steroids (Cherry and Glucksmann, 1960 ; Glucksmann and Cherry, 1958). In the mouse there is a distinct parallelism between the sensitivity of the cervico-vaginal epithelium to benzo(a)pyrene and the response of the epidermis (Glucksmann, 1945 ; Berenblum, 1945).

The sarcogenic effect of the chemical carcinogens seems in the rat to be a genetic species characteristic.* This tendency to sarcogenesis is also revealed by the spontaneous tumours. Indeed, for spontaneous tumours in the rat the ratio of sarcoma to carcinoma is high (2 : 1) and in the mouse very low (1 : 5) (Tamaschke, 1955). It seems, therefore, as though in the former species the connective tissue is particularly sensitive to exogenic and endogenic tumorigenic noxa.

In the hamster we failed to induce sarcoma in the genital tract. None the less, it is known that in this species sarcoma can be provoked without difficulty by subcutaneous injection of benzo(a)pyrene (Gye and Foulds, 1939 ; Halberstaedter, 1940) or DMBA (Crabb, 1946). Two *Mastomys* (2/10) developed sarcoma of the vaginal wall.

Lastly, a word about the coincidental pathological lesions. In painted mice, infectious processes in and outside the genital tract (peritonitis ; abscesses of the liver, lung, and small intestine) are frequent, which is possibly to be ascribed to the treatment. Splenomegaly is often found in animals with large cervical tumours. The weight of the spleen of C3H mice which had been painted 30–38 times was 0·125 to 0·824 g., averaging 0·413 g. ; that of the control animals of comparable age was 0·177–0·411 g., averaging 0·240 g. This difference in weight between the treated and untreated animals is highly significant ($P<0 \cdot 001$). The explanation of splenomegaly in animals with spontaneous and experimentally-induced cancer is not evident (Hoepke, 1953, 1955), but it is known that hypertrophy of this organ is also found in patients with carcinoma of the uterine cervix (Meigs, 1954).

Extrinsic pressure exerted by the tumour plays a role in the development of hydro-ureteronephrosis, muco(pyo)metra, and muco(pyo)salpinx. The following genital tumours were observed : cystoma ovarii simplex, serous cyst of the tube, and teratoma ovarii solidum. The first two tumours were incidental findings, but for the last-named an aetiological relationship with the carcinogen employed is suspected (Thiery, 1963*b*). The following extragenital tumours were found : mammary carcinoma and pulmonary tumours. In painted C3H mice the incidence of breast cancer is low (3 %) but comparable with that of the control virgins of the same age group (Boot and Muhlbock, 1956) ; thus, it may be concluded that painting of the portio with benzo(a)pyrene has no influence on the incidence

* The rat is the most sensitive of all the common laboratory rodents to subcutaneous injection of benzo(a)pyrene, to which it reacts by the formation of sarcomas (Berenblum, 1945, 1949).

of mammary carcinoma in this strain. The probably-significant frequent occurrence of mucoepidermoid carcinoma in mice with mammary carcinoma is remarkable ($0 \cdot 1 < P < 0 \cdot 05$). Pulmonary adenoma was found in 4 mouse strains (N, Q, AKR, H) with a cumulative incidence of 7 %. The highest incidence was found in AKR mice (4/10). No tumours of this type were observed in C3H mice. Spontaneous adenoma of the lung has been described for hybrid mice (2 to 5·7 % : Lippincott *et al.*, 1942 ; Wells, Slye and Holmes, 1941), but occurs almost exclusively in old animals. This tumour has never been found in untreated AKR mice (Muhlbock, 1962, personal communication), so that benzo(a)pyrene may be responsible for its occurrence. One H mouse showed not only adenoma in the lung but also adenocarcinoma ; the latter tumour had in all probability metastasized in the liver. The other rodent species showed no unusual coincidental pathologic lesions. Adenoma of the lung was found only in *Mastomys* (1/10).

### SUMMARY

In mice belonging to 12 different strains (C3H, A, 0·20, S, WLL, AKR, Q, N, R, K, H, and Mo), rats, hamsters, guinea-pigs, and *Mastomys natalensis*, the portio was visually painted twice a week with a suspension of 1% 3,4-benzo(a)-pyrene in acetone.

Although squamous cell carcinoma of the portio and/or vagina was induced in all the mouse strains, distinct strain differences in the neoplastic response of the cervico-vaginal squamous epithelium to benzo(a)pyrene were observed.

The other rodents also showed pronounced differences according to species in the response of the epithelium of the portio and vagina. Hamsters are extremely sensitive to benzo(a)pyrene. The response of *Mastomys natalensis* is less conspicious ; in rats and guinea-pigs epithelial carcinogenesis failed.

The induced cervico-vaginal tumours belonged to the squamous cell type. A variant, mucoepidermoid carcinoma, is not infrequent in the mouse, hamster, and *Mastomys natalensis*. Sarcoma of the vaginal walls and portio is rare in treated mice. In *Mastomys natalensis* and in the rat the sarcogenic effect of 3,4-benzo(a)-pyrene is more pronounced.

The following coincidental pathological lesions were observed : genital and extra-genital infections, splenomegaly, cysts of the ovary and tube. The incidence of mammary carcinoma in painted C3H mice does not differ from that of the controls. For the genesis of ovarian teratoma and pulmonary tumours, the aetiological role of benzo(a)pyrene cannot be excluded. Adenoma of the lung was seen in four mouse strains (N, Q, AKR, and H) ; its occurrence is possibly to be ascribed to the influence of the carcinogen employed.

This work was supported by a grant from the National Fonds voor Wetenschappelijk Onderzoek and the Fonds voor Wetenschappelijk Geneeskundig Onderzoek.

The authors wish to acknowledge the valuable technical assistance provided by Mrs. M. A. Vercauteren and Miss E. van Overbeke.

### REFERENCES

Berenblum, I.—(1945) *Cancer Res.*, **5**, 561.—(1949) *J. nat. Cancer Inst.*, **10**, 167.

Bogacz, J. and Koprowska, I.—(1961) *Acta Cytol.*, **5**, 311.

BOOT, L. M. AND MUHLBOCK, O.—(1956) *Acta Un. int. Cancr.*, **12**, 569.
BULLOCK, F. D. AND CURTIS, M. R.—(1930) *J. Cancer Res.*, **14**, 1.
CARTER, T. C., DUNN, L. C., FALCONER, D. S., GRUNEBERG, H., HESTON, W. E. AND SNELL, G. D.—(1952) *Cancer Res.*, **12**, 602.
CHERRY, C. P. AND GLUCKSMANN, A.—(1960) *Brit. J. Cancer*, **14**, 489.
CHU, E. W., HERROLD, K. Mc.D. AND WOOD, T. A.—(1962) *Acta Cytol.*, **6**, 376.
CRABB, E. D.—(1946) *Cancer Res.*, **6**, 627.
VON EULER, H. AND SKARZYNSKI, B.—(1942) 'Biochemie der Tumoren'. Stuttgart (F. Enke Verlag).
FUSCO, G.—(1932) *Arch. Ostet. Ginec.*, **19**, 315, 467.
GLUCKSMANN, A.—(1945) *Cancer Res.*, **5**, 385.—(1956) *Rep. Brit. Emp. Cancer Campgn*, **34**, 242.
*Idem* AND CHERRY, C. P.—(1958) *Brit. J. Cancer*, **12**, 32.—(1962) *Ibid.*, **16**, 634.
GYE, W. E. AND FOULDS, L.—(1939) *Amer. J. Cancer*, **35**, 108.
VON HAAM, E. AND ALBERTY—(1960) cited by von Haam E. and Scarpelli. D. G. (1960) in 'Progr. Exp. Tumor Res.' New York (Karger). Vol. I, pp. 179–224.
*Idem* AND SCARPELLI, D. G.—(1955) *Cancer Res.*, **15**, 449.
HALBERSTAEDTER, L.—(1940) *Amer. J. Cancer*, **38**, 351.
HOEPKE, H.—(1953) *Verh. dtsch. path. Ges.*, **37**, 202.—(1955) *Mikroskopie*, **10**, 268.
IBALL, J.—(1939) *Amer. J. Cancer*, **35**, 188.
KOPROWSKA, I., BOGACZ, J., PENTIKAS, C. AND STYPULKOWSKI, W.—(1958) *Cancer Res.*, **18**, 1186.
KORTEWEG, R. AND WIJSENBEEK, I. A.—(1933) *Ned. Tijdschr. Geneesk.*, **77**, 4069.
LIPPINCOTT, S. W., EDWARDS, J. E., GRADY, H. G. AND STEWART, H. L.—(1942) *J. nat. Cancer Inst.*, **3**, 199.
MEIGS, J. V.—(1954) 'Surgical treatment of cancer of the cervix'. New York (Grune & Stratton).
MURPHY, E. D.—(1961) *J. nat. Cancer Inst.*, **27**, 611.
OBERLING, CH. AND GUERIN, M.—(1947) *Bull. Ass. franc. Cancer*, **34**, 30.
*Idem*, SANNIE, C., GUERIN, M. AND GUERIN, P. (1936) *Ibid.*, **25**, 156.
PAN, S. C. AND GARDNER, W. U.—(1948) *Cancer Res.*, **8**, 613.
REAGAN, J. W., WENTZ, W. B. AND MACHICAO, N.—(1955) *Arch. Path.*, **60**, 451.
SCARPELLI, D. G. AND VON HAAM, E.—(1957) *Amer. J. Path.*, **33**, 1059.
SILBERSTEIN, M. AND SILBERSTEIN, R.—(1947) *Arch. Path.*, **43**, 143.
TAMASCHKE, C.—(1955) *Strahlentherapie*, **96**, 150.
THIERY, M.—(1963*a*) 'Het Experimentele Carcinoma Colli Uteri.' Brussels (Arscia & Presses Académiques Européennes).—(1963*b*) *Acta Cytol.*, **7**, 72.
THOMAS, F.—(1935) *Acta brev. neerl. Physiol.*, **4**, 179.—(1936) *Arch. int. Pharmacodyn.*, **53**, 65.
VELLIOS, F. AND GRIFFIN, J.—(1957) *Cancer Res.*, **17**, 364.
WACHTEL, E.—(1956) *Rep. Brit. Emp. Cancer Campgn*, **34**, 222.—(1961) *J. Obstet. Gynaec. Brit. Commonw.*, **68**, 101.
WELLS, H. G., SLYE, M. AND HOLMES, H. F.—(1941) *Cancer Res.*, **1**, 259.


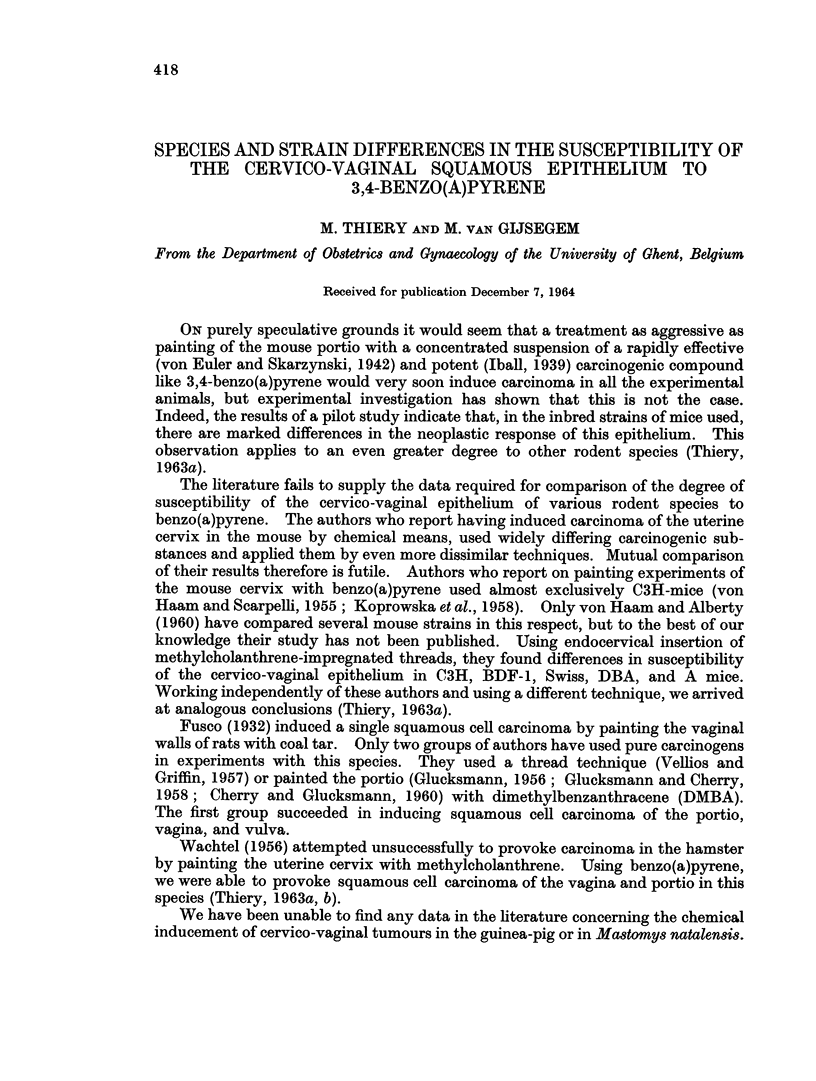

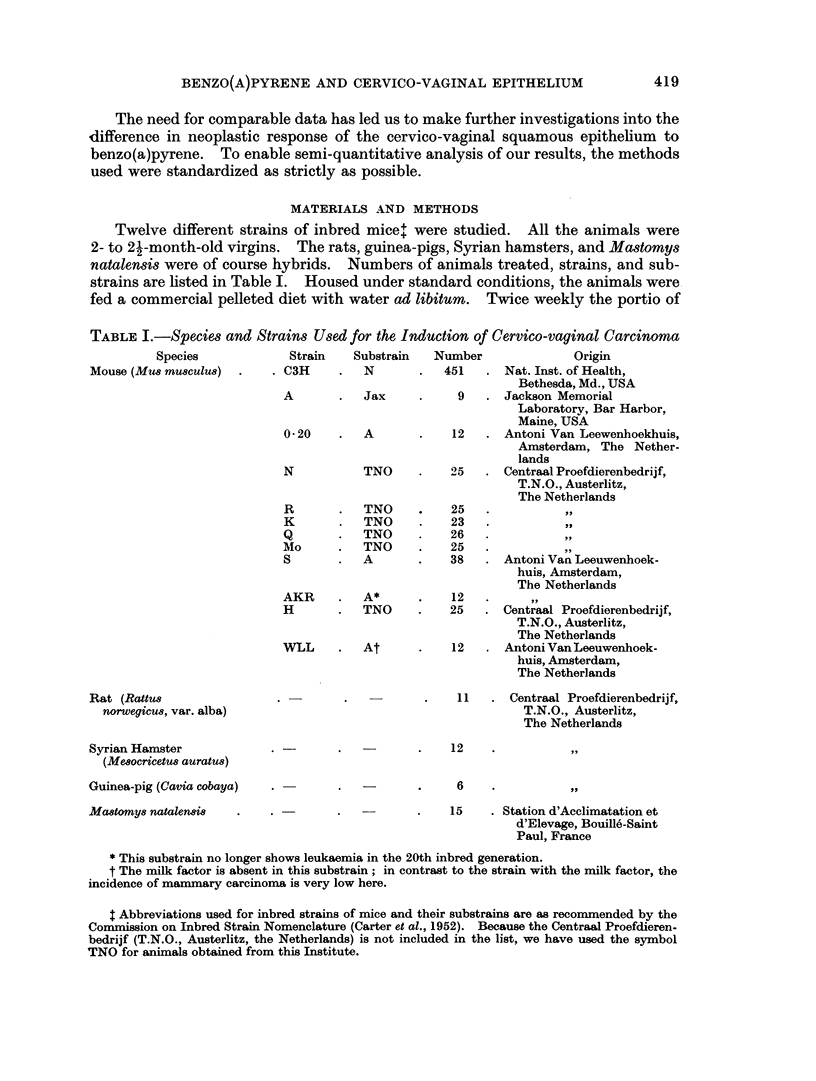

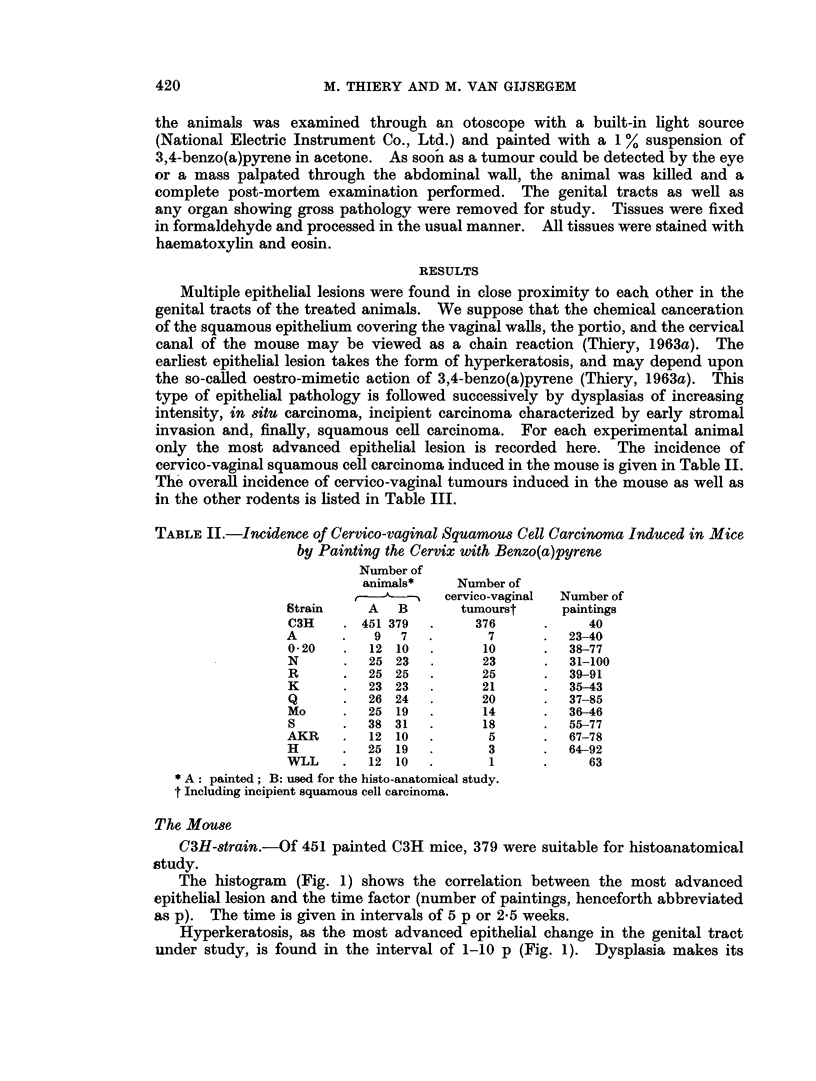

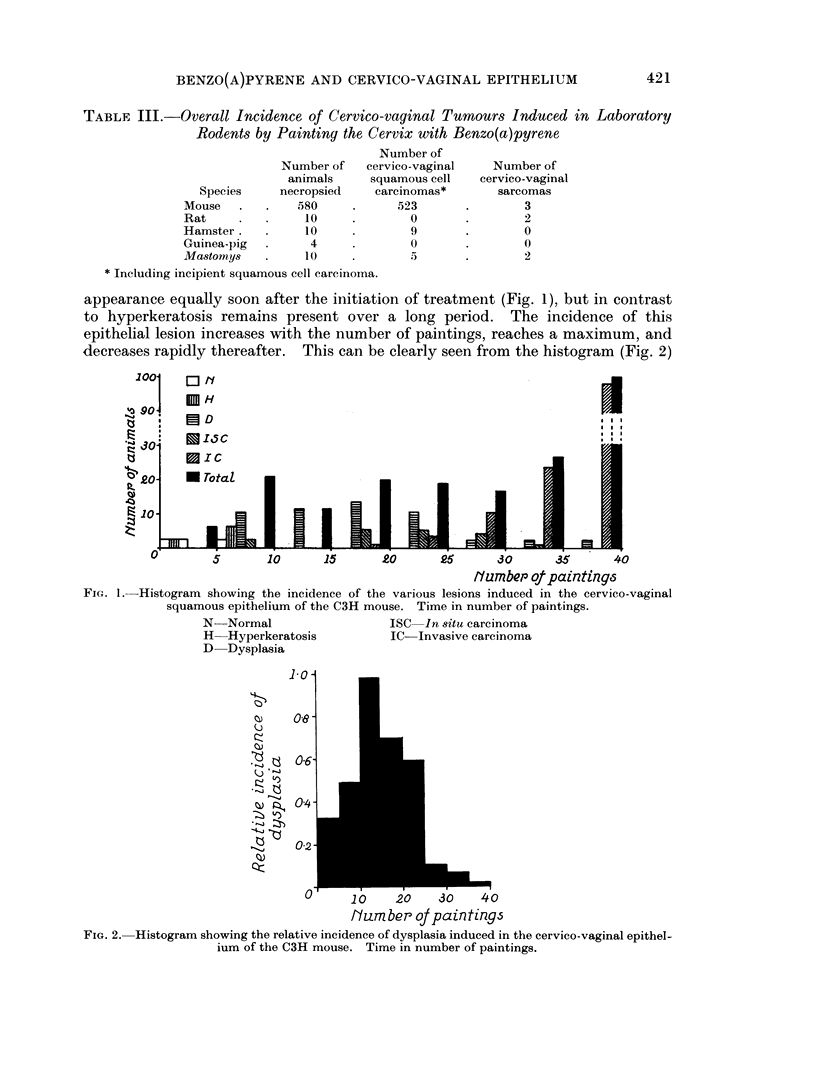

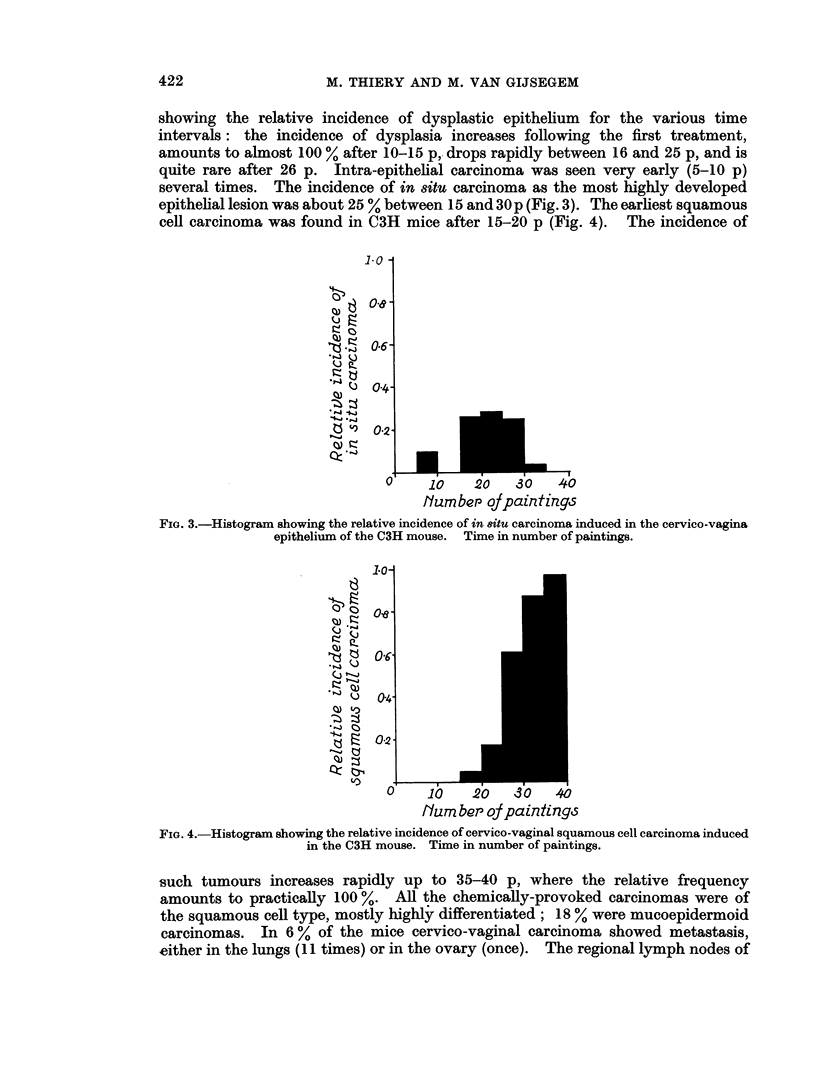

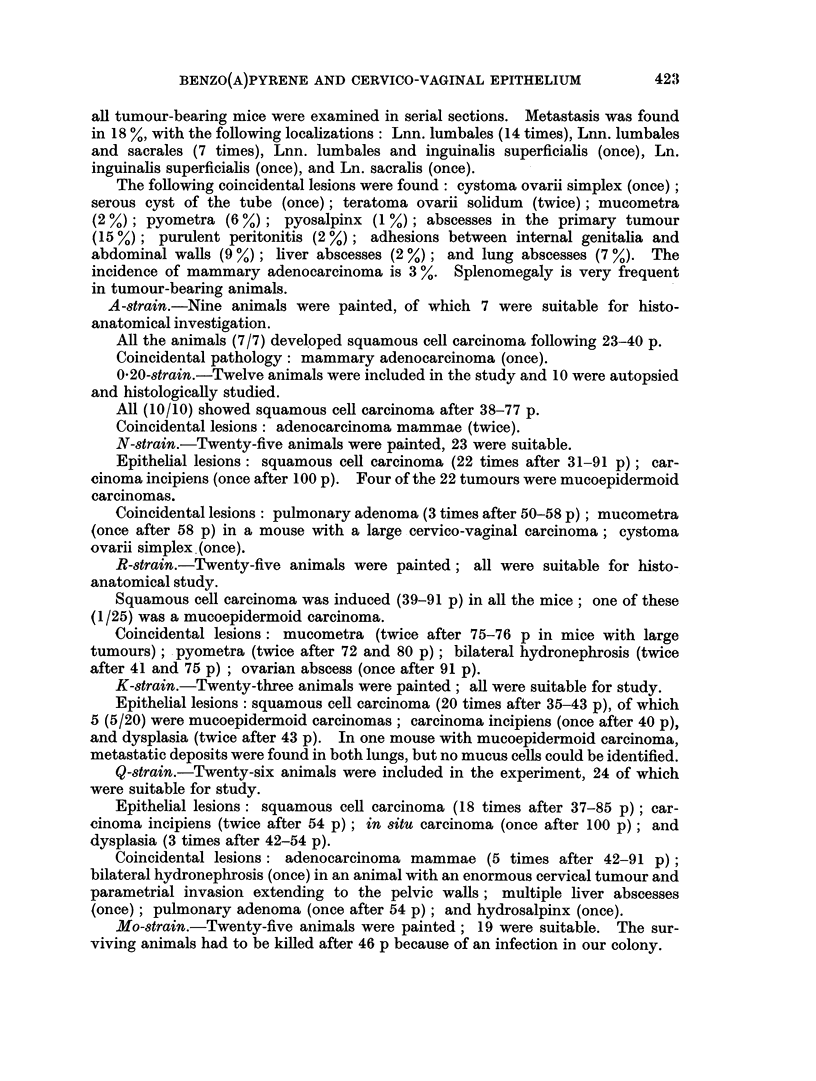

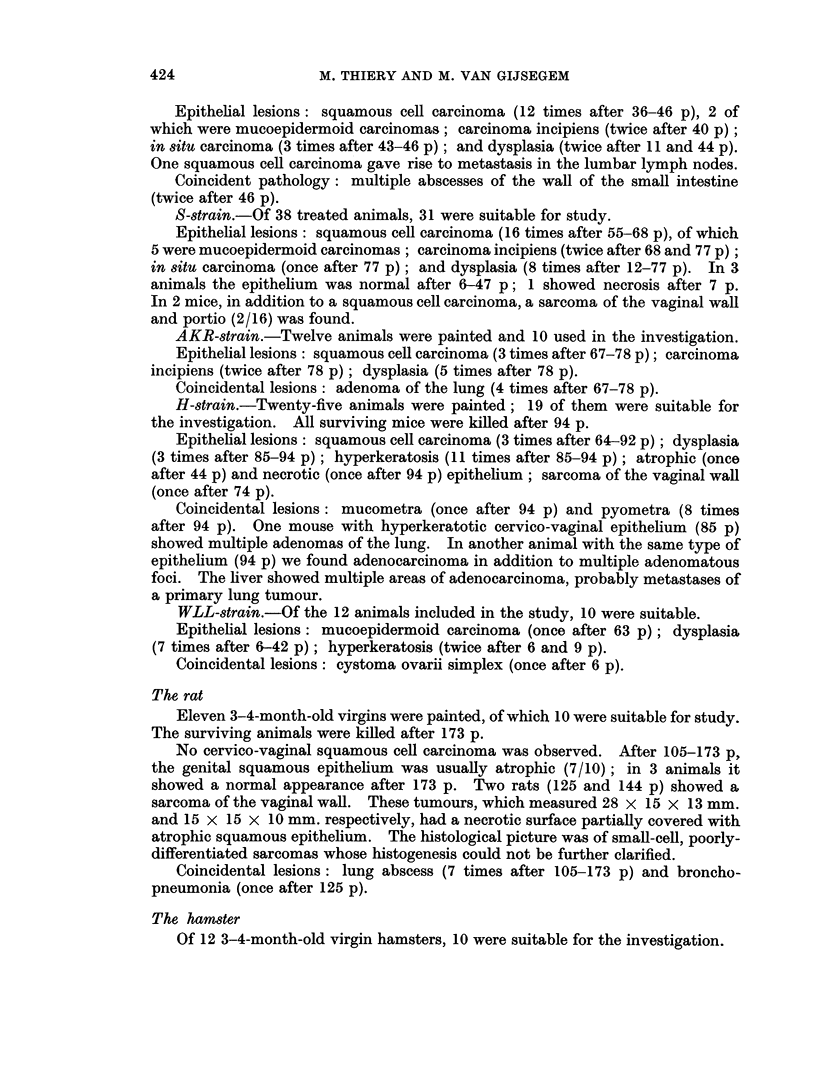

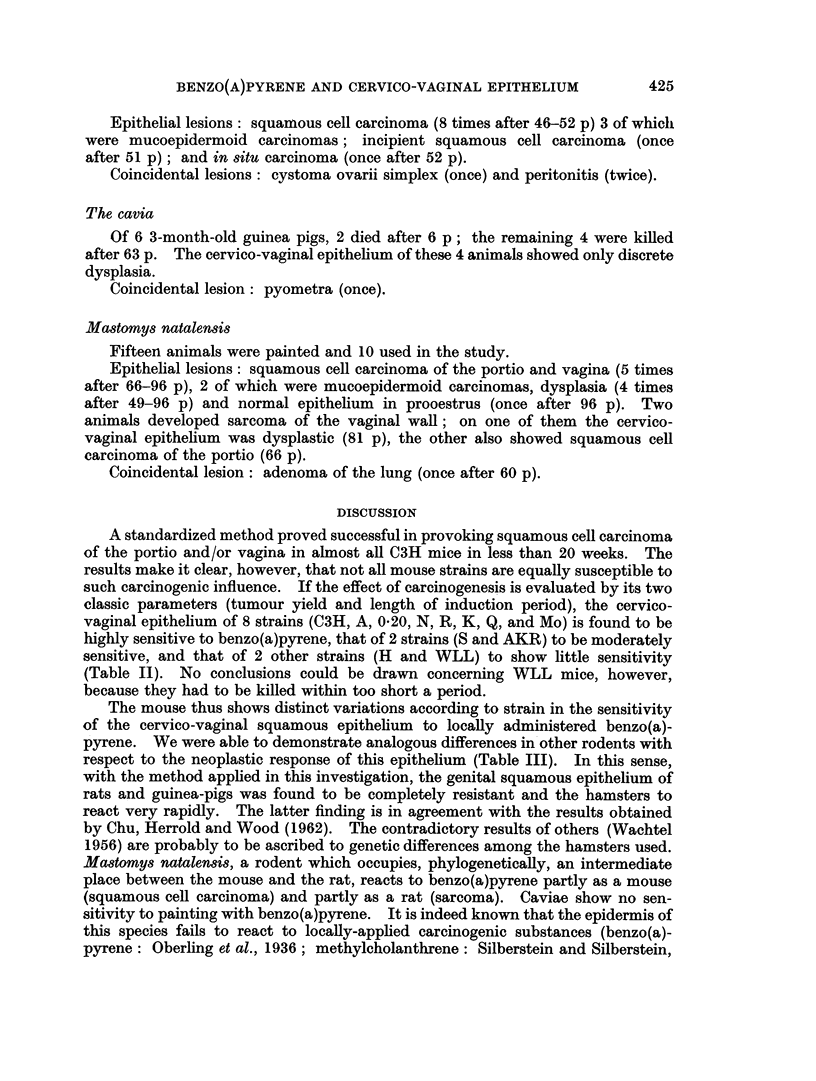

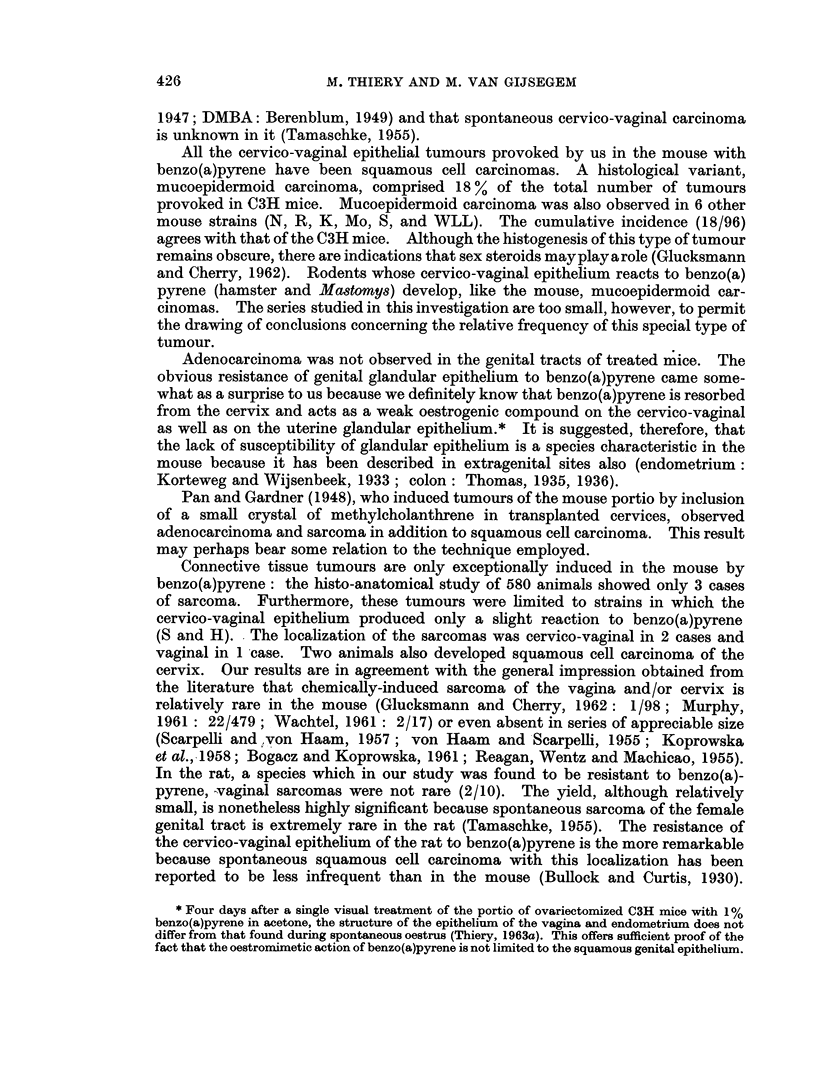

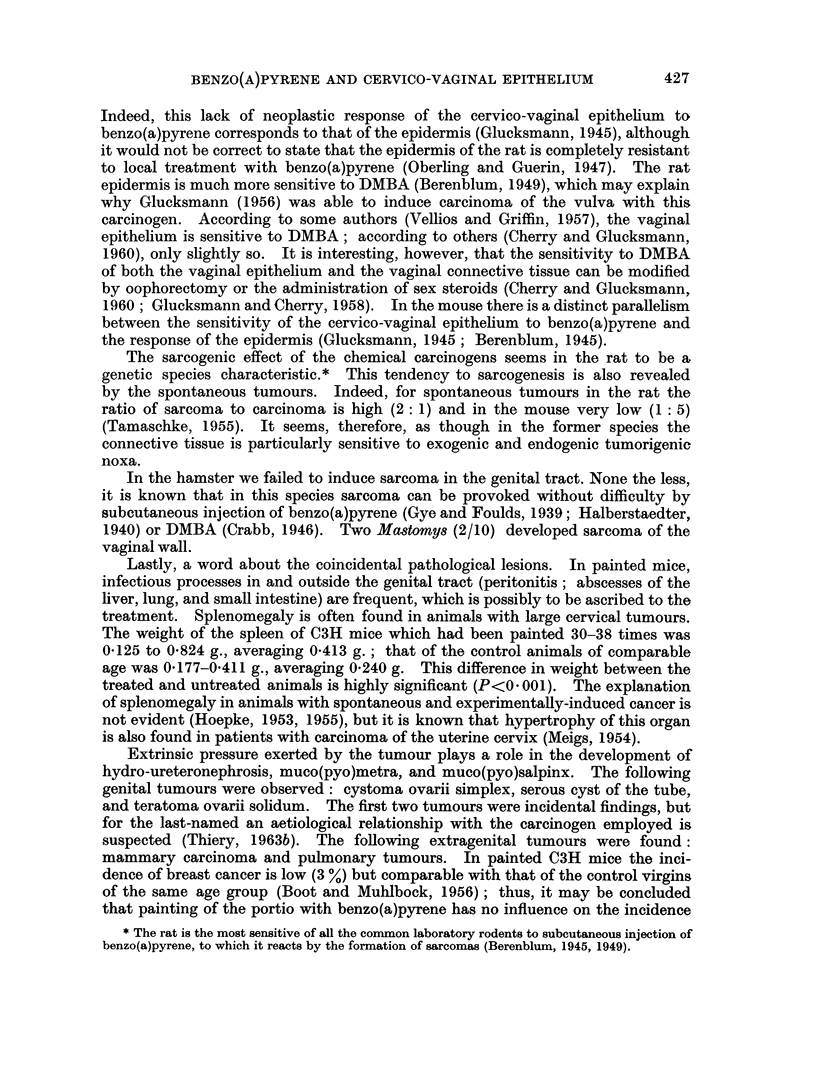

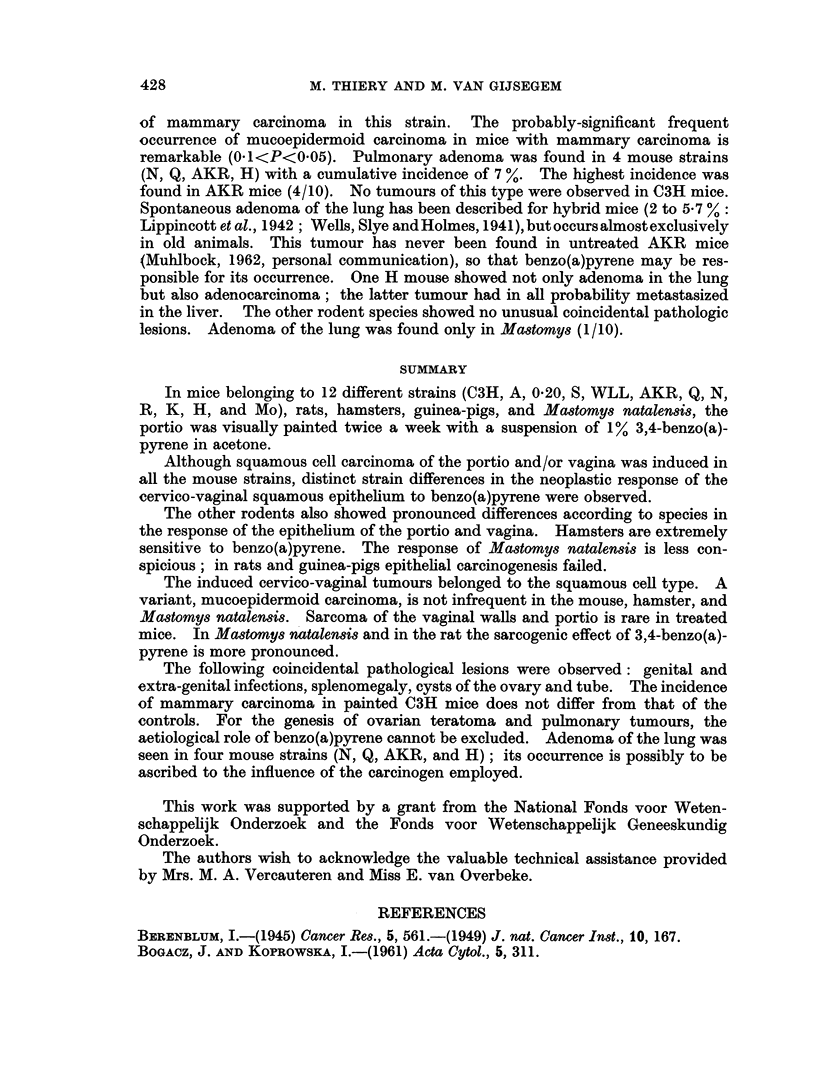

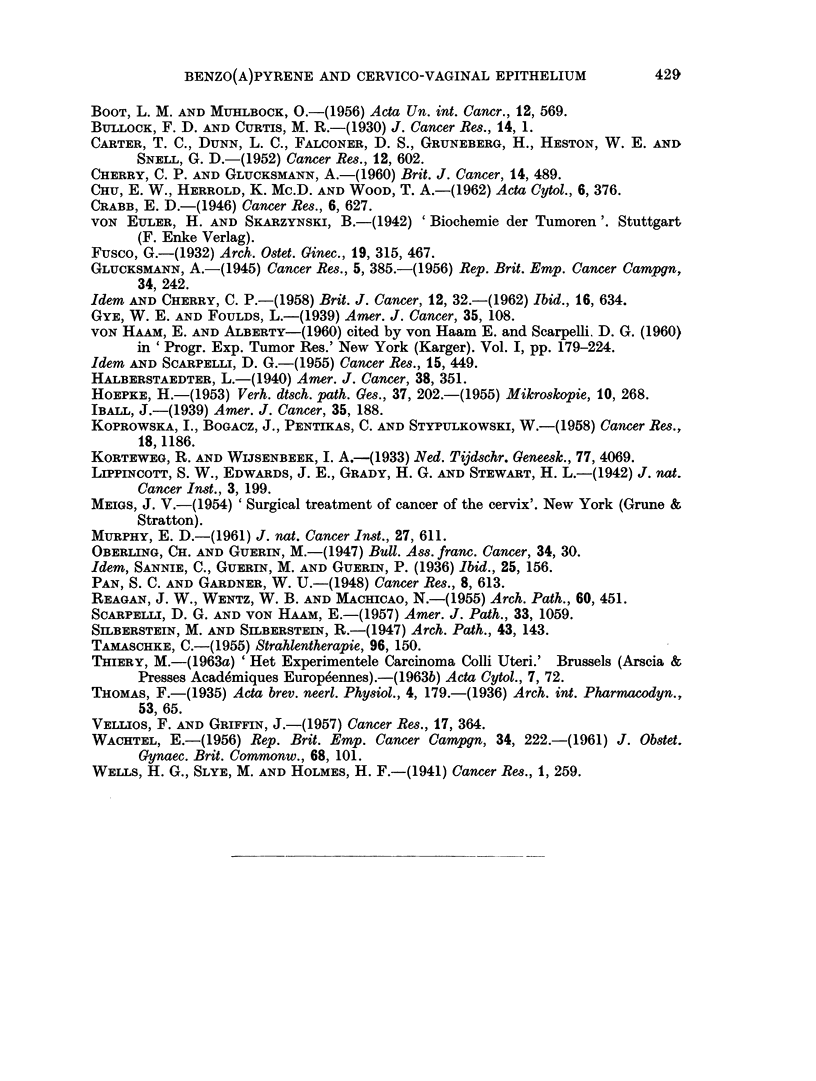


## References

[OCR_00762] BOOT L. M., MUHLBOCK O. (1956). The mammary tumour incidence in the C3H mouse-strain with and without the agent (C3H; C3Hf; C3He).. Acta Unio Int Contra Cancrum.

[OCR_00771] CHU E. W., HERROLD K. M., WOOD T. A. (1962). Cytopathological changes of the uterine cervix of Syrian hamsters after painting with DMBA, benzo(a)pyrene, and tobacco tar.. Acta Cytol.

[OCR_00794] HOEPKE H. (1956). Die Reaktion von Milz und Thymus beim spontanen Mammakarzinom der Maus.. Mikroskopie.

[OCR_00796] KOPROWSKA I., BOGACZ J., PENTIKAS C., STYPULKOWSKI W. (1958). Induced cervical carcinoma of the mouse; a quantitative cytologic method for evaluation of the neoplastic process.. Cancer Res.

[OCR_00812] MURPHY E. D. (1961). Carcinogenesis of the uterine cervix in mice: effect of diethylstilbestrol after limited application of 3-methylcholanthrene.. J Natl Cancer Inst.

[OCR_00817] SCARPELLI D. G., VON HAAM E. (1957). Experimental carcinoma of the uterine cervix in the mouse; a gross and histopathologic study.. Am J Pathol.

[OCR_00789] SCARPELLI D. G., von HAAM (1960). Experimental carcinoma of the uterine cervix.. Prog Exp Tumor Res.

[OCR_00819] TAMASCHKE C. (1955). Die Spontantumoren der kleinen Laboratoriumssäuger in ihrer Bedeutung für die experimentelle Onkologie.. Strahlentherapie.

[OCR_00829] VELLIOS F., GRIFFIN J. (1957). The pathogenesis of dimethylbenzanthracene-induced carcinoma of the cervix of rats.. Cancer Res.

[OCR_00790] VON HAAM E., SCARPELLI D. G. (1955). Experimental carcinoma of the cervix: a comparative cytologic and histologic study.. Cancer Res.

[OCR_00831] WACHTEL E. (1961). Experimental cancer of the uterus in C3H strain mice.. J Obstet Gynaecol Br Commonw.

